# Primary intestinal lymphangiectasia diagnosed by double-balloon enteroscopy and treated by medium-chain triglycerides: a case report

**DOI:** 10.1186/1752-1947-7-19

**Published:** 2013-01-14

**Authors:** Yu Lai, Tao Yu, Xiao-yu Qiao, Li-na Zhao, Qi-kui Chen

**Affiliations:** 1 Department of Gastroenterology, Sun Yat-sen Memorial Hospital, Sun Yat-Sen University, 107 Yan Jiang Xi Road, Guangzhou, Guangdong 510120, People’s Republic of China; 2 Department of Emergency, the Third Affiliated Hospital, Sun Yat-sen University, Guangzhou, Guangdong, People’s Republic of China

**Keywords:** Diarrhea, Edema, Hypoproteinemia, Intestinal lymphangiectasia, Medium-chain triglyceride diet

## Abstract

**Introduction:**

Primary intestinal lymphangiectasia is a disorder characterized by exudative enteropathy resulting from morphologic abnormalities of the intestinal lymphatics. Intestinal lymphangiectasia can be primary or secondary, so the diagnosis of primary intestinal lymphangiectasia must first exclude the possibility of secondary intestinal lymphangiectasia. A double-balloon enteroscopy and biopsy, as well as the pathology can be used to confirm the diagnosis of intestinal lymphangiectasia. A polymeric diet containing medium-chain triglycerides and total parenteral nutrition may be a useful therapy.

**Case presentation:**

A 17-year-old girl of Mongoloid ethnicity was admitted to our hospital with a history of diarrhea and edema. She was diagnosed with protein-losing enteropathy caused by intestinal lymphangiectasia. This was confirmed by a double-balloon enteroscopy and multi-dot biopsy. After treatment with total parenteral nutrition in hospital, which was followed by a low-fat and medium-chain triglyceride diet at home, she was totally relieved of her symptoms.

**Conclusion:**

Intestinal lymphangiectasia can be diagnosed with a double-balloon enteroscopy and multi-dot biopsy, as well as the pathology of small intestinal tissue showing edema of the submucosa and lymphangiectasia. Because intestinal lymphangiectasia can be primary or secondary, the diagnosis of primary intestinal lymphangiectasia must first exclude the possibility of secondary intestinal lymphangiectasia. A positive clinical response to the special diet therapy, namely a low-fat and medium-chain triglyceride diet, can further confirm the diagnosis of primary intestinal lymphangiectasia.

## Introduction

Primary intestinal lymphangiectasia (PIL), also named Waldmann’s disease, is a rare disorder characterized by exudative enteropathy resulting from morphologic abnormalities of the intestinal lymphatics [1]. Edema (moderate to severe with pleural effusion, pericarditis, or chylous ascites) is the main clinical manifestation but lymphedema, fatigue, abdominal pain, weight loss, moderate diarrhea, and fat-soluble vitamin deficiencies may also be present [1]. Patients can also develop hypocalcemia secondary to failure to absorb fat and fat-soluble vitamins.

It is difficult to confirm the diagnosis of PIL using serological tests, endoscopy, colonoscopy and normal image check, but the diagnosis can be established by double-balloon enteroscopy, multi-dot biopsy, and especially by the pathologic examination of small intestinal tissue showing edema of the submucosa and lymphangiectasia. Intestinal lymphangiectasia (IL) can be primary or secondary so the diagnosis of PIL must first exclude the possibility of secondary IL. Tests that exclude proteinuria and rheumatic, neoplastic and parasitic infection can be used to exclude the possibility of secondary IL.

Patients who have been discharged with an improvement in hypoproteinemia may prevent recurrent enteric protein loss by using total parenteral nutrition (TPN) and medium-chain triglycerides (MCTs). The need for fat restriction has been described as permanent due to the frequent clinical relapses upon relaxation of the regimen. It is considered that, when treating out-patients with IL, enteral nutrition should be continued as long as hypoproteinemia is present [2,3]. In more severe cases, the use of octreotide, a synthetic analogue of the naturally occurring hormone somatostatin (a potent inhibitor of the release of growth hormone, serotonin, gastrin, glucagon and insulin), has been found to be successful [4].

Here, we present a case of PIL that was diagnosed by double-balloon enteroscopy and multi-biopsy, and had a positive response to TPN and MCTs.

## Case presentation

A 17-year-old girl of Mongoloid ethnicity presented to our hospital with a history of diarrhea and edema in her eyelids and lower limbs for three weeks, which were secondary to a transient fever lasting one week. About one month prior to this admission, the patient complained of a fever (the highest temperature being 39°C) accompanied by sore throat, cough, and runny nose, but no fear of cold or shivering. These symptoms lasted for about one week and disappeared. Then the diarrhea and edema in her eyelids and lower limbs began. The frequency of stools was about two to three times a day. The stools were like yellow water with a little foam, and contained undigested foods. The patient also complained of poor appetite, occasional abdominal distention and abdominal pain. Her food intake was reduced to between one-third and half of her usual amount. She usually had gruel and did not like meat. She lost two kg in this one month. She denied hair loss, rash, skin erythema, oral ulcers, photosensitivity, joints pain, foamy urine, palpitations, night sweats, headache, chest pain, paroxysmal nocturnal dyspnea, insomnia, irritability, and tremor. There was nothing of significance in her medical history or drug therapy.

Her physical examination demonstrated malnutrition and edema in her eyelids and lower limbs. A few lymph nodes of 0.5×0.5cm in size were found in her submandibular area, in her axillas and inguina on both sides, with clear borders and no tenderness. Her body mass index was 15kg/m^2^. Her heart rate was 120 beats per minute and other systematic examinations revealed no positive signs.

She was admitted to the Rheumatology department of our hospital at first. Investigations showed decreased white blood cells (2.71×10^9^/L; neutrophils 1.64×10^9^/L, lymphocytes 0.34×10^9^/L), anemia (hemoglobin 100g/L), hypoalbuminemia (serum albumin 20.5g/L), hypoimmunoglobulinemia (globulin 12g/L), hypolipoproteinemia (cholesterol 2.8mmol/L; triglyceride 0.28mmol/L), and hypocalcemia (1.52mmol/L). During her hospital stay, the patient had numbness and convulsions in both hands several times and these symptoms were relieved by calcium and vitamin D therapy. The results of a Coombs’ test and anticardiolipin antibodies were both negative. Results of tests related to Sjögren’s syndrome, systemic lupus erythematosus, rheumatoid arthritis, and vasculitis were almost normal. The results of the investigation indicated that rheumatologic disease could be ruled out.

Further investigations showed mildly increased free triiodothyronine, anti-thyroid-stimulating hormone receptor antibody, parathyroid hormone, phosphorus, and decreased thyroid-stimulating hormone in serum. Emission computed tomography showed a diffuse goiter, and an increased thyroid technetium uptake function. Ultrasound showed ascites of 32.6mm in depth. The patient was transferred to the Department of endocrinology.

The patient had paroxysmal paralysis in her palms many times. The attacks were always accompanied by the hyperextension of her fingers. Each attack lasted a few minutes. The symptoms could be relieved by the intravenous injection of calcium gluconate. The serium calcium levels fluctuated in the range of 1.33 to 1.52mmol/L. After the patient was given calcium and vitamin D supplementation, the symptoms stopped. Her 24-hour urinary protein excretion and lymphangiography were both normal. The Epstein–Barr virus and hepatitis virus were not detected in her serum. An electronic gastroscope revealed superficial antral gastritis. Capsule endoscopy showed that villi edema were present in the small intestinal (descending part of the duodenum and upper jejunum part of duodenum) mucosa. So at last she was transferred to the Department of gastroenterology.

**Figure 1:**
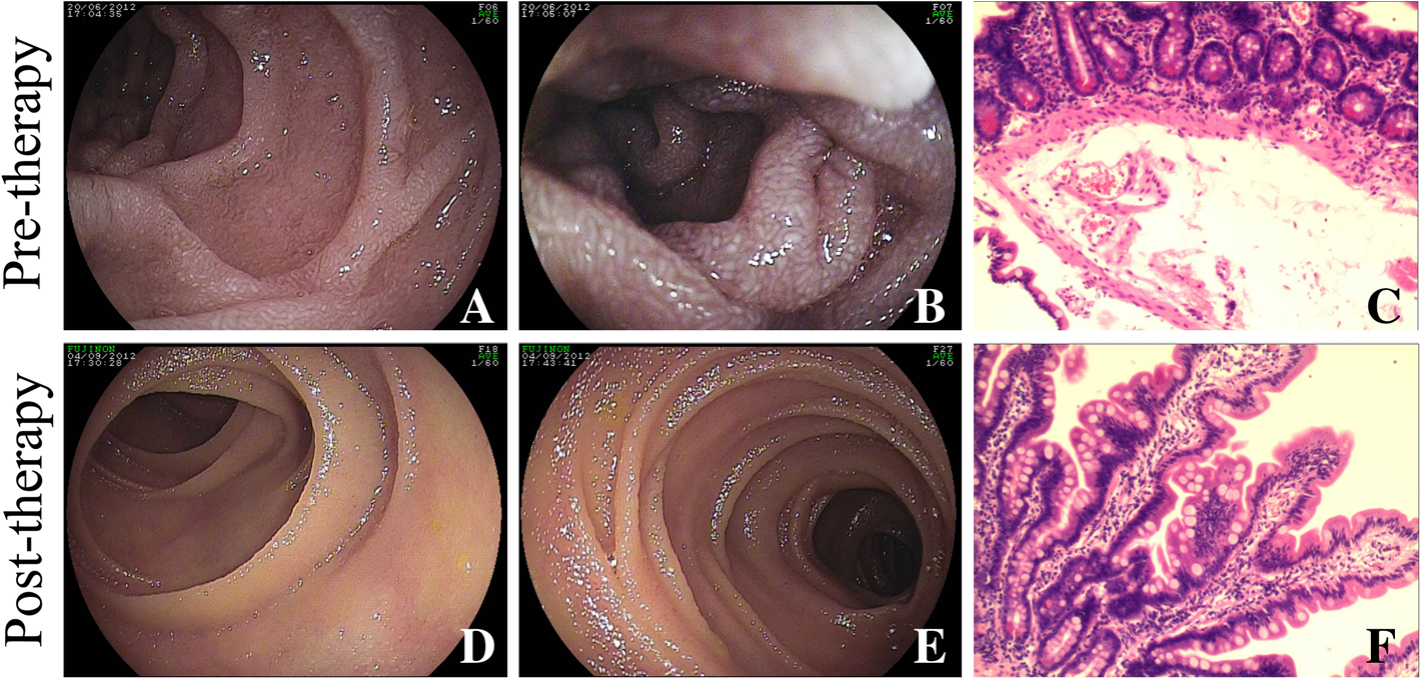
**A double-balloon enteroscopy and pathologic investigation were performed before and after 10-week treatment. A** and **B**: The double-balloon enteroscopy revealed lymphangiectasia-like changes in the small intestinal mucosa. **C**: The pathologic analysis of small intestinal tissue before treatment showed chronic inflammation, edema in the submucosa, and lymphangiectasia (hematoxylin and eosin stain×200). **D** and **E**: The lymphangiectasia-like changes in the small intestinal mucosa vanished after diet therapy. **F**: The pathologic results after treatment were improved (hematoxylin and eosin stain×200).

A double-balloon enteroscopy revealed lymphangiectasia-like changes in the small intestinal mucosa (descending part of the duodenum and upper jejunum part of duodenum; Figure 1A, 1B). The pathologic analysis of small intestinal tissue showed chronic inflammation, edema in the submucosa, and lymphangiectasia (Figure 1C).

Based on these findings, a therapy composed of albumin and calcium supplementation and parenteral nutrition support was given to the patient. The patient undertook recipes that strictly conformed to the low-fat and MCT diet. The daily intake of cooking oil on MCTs was 25 g and the total fat intake must not exceed 40g/day.

After 10 weeks of dietary treatment, the patient’s clinical manifestations and nutritional status were improved. The patient no longer felt any discomfort in her daily life and work. Results of the laboratory examination conducted after the 10-week diet therapy are shown in Table 1. A repeat double-balloon enteroscopy showed that the lymphangiectasia-like change in the small intestines had vanished. The double-balloon enteroscopy results are shown in Figure 1D and 1E, and the pathology is shown in Figure 1F.

**Table 1:** Results of the laboratory examination conducted before and after the 10-week diet therapy

**Laboratory examination**	**Pre-treatment**	**After the 10-week diet therapy**
White blood cell	2.71×10^9^/L	4.02×10^9^/L
neutrophils	1.64×10^9^/L	2.56×10^9^/L
lymphocytes	0.34×10^9^/L	1.08×10^9^/L
Hemoglobin	100 g/L	110 g/L
Serum albumin	20.5 g/L	32.5 g/L
Serum globulin	12 g/L	22 g/L
Serum calcium	1.52 mmol/L	2.05 mmol/L
Serum cholesterol	2.8 mmol/L	5.1 mmol/L
Serum triglyceride	0.28 mmol/L	1.00 mmol/L

## Discussion

IL is a relatively rare disorder that manifests itself through intestinal malabsorption. Its worldwide incidence and prevalence is unknown. The disease affects males and females equally, and there is no racial predilection. The disease can be primary (congenital), where there is malformation within the lymphatic channels leading to their blockage, and the condition is usually diagnosed within the first decade of life. The condition can also be secondary to other disease states, such as constrictive pericarditis, lymphoma, sarcoidosis and scleroderma, and it can affect older adults. The first manifestations are usually persistent diarrhea and peripheral edema. The edema can be unilateral or bilateral, and macular edema revealed in funduscopic examination has been reported (blindness caused by this is reversible). Common symptoms of IL are steatorrhoea, lymphocytopenia, hypogammaglobulinemia, hypoproteinemia, and malabsorption [5]. The mechanisms for enteric protein loss in IL are not well understood, although an increase in the pressure of the lymph channels has been suggested to be a possible cause of protein loss [2,3].

A jejunal biopsy usually establishes a definitive diagnosis, and displays dilation of mucosal and submucosal lymphatic channels without any evidence of inflammation. The lymphatic hypoplasia results in an obstruction in lymph flow, which leads to increased pressure within the lymphatics. This, in turn, will cause dilation of the lymphatic channels in the intestine and, finally, lead to the rupture of the channels and resultant discharge of the lymph into the lumen of the bowel [5]. The characteristic endoscopic and radiographic features of IL have been documented thereby greatly facilitating an accurate diagnosis [6,7].

In this case, the first presentation was found to be hypoproteinemia. After excluding synthetic obstacles and insufficient intake as the causes, we concluded that protein loss was the main factor. We also repeated some tests to exclude proteinuria. At last, we were sure that it was a protein-losing gastrointestinal disease. Thus we carried out a gastroscopy, colonoscopy and capsule endoscopy to locate the lesion, and used a double-balloon endoscope to perform the biopsy in the lesion of IL, based on some tests that excluded rheumatic, neoplastic, and parasitic infection. The diagnosis of PIL was established.

Enteral nutrition, as well as TPN, may be a useful therapy in patients with IL. Once the serum protein level has returned to normal, continuation of enteral nutrition appears to be valuable in preventing relapse. Compared with TPN, the elemental diet and the polymeric diet, which contains MCTs, are ideal maintenance therapies because they are safer, inexpensive, and more convenient to prepare at home [8]. The main clinical manifestation of this case was severe malnutrition and, following this, the appearance of hypoproteinemia, hypocalcemia, and hypothyroxinemia. However, the complaint of diarrhea was not as serious. Based on these findings, a therapy composed of albumin and calcium supplementation and parenteral nutrition support was given to the patient.

In this case TPN was used at first so that the patient could recover quickly from hypothyroxinemia, hypocalcemia, and hypoproteinemia. However, TPN is an inconvenient therapy, so we chose enteral nutrition which contains a low-fat diet and a MCT diet. There has been no specific therapy for this condition, although it is known that fat restriction can successfully reduce gastrointestinal protein loss in most patients with IL. Surgery may be successful when fibrotic changes of the small bowel cause partial mechanical bowel obstruction [9]. Antiplasmin therapy has been applied to patients who respond poorly to other therapies [10]. Alternatively, octreotide has been documented to improve protein-losing enteropathy in IL by speculative mechanisms including reduction in lymph flow and immunomodulatory action [11,12]. When patients do not respond to a fat-restricted diet, these medical therapies should be considered.

Because a partial block of the intestinal lymphatic system produces a loss of lymph into the lumen upon a high-fat diet, dietary fat restriction is considered to be the first choice of treatment in IL [2,13,14]. In practice, reduction of the intestinal lymph flow through fat restriction has been reported [2,3]. Likewise, a diet containing MCTs as a substitute for long-chain triglycerides is of benefit because MCTs are absorbed directly into the portal vein, rather than the lymphatics, thereby reducing the pressure of the lymph channels [15].

We preferred to choose the special diet as the first therapy. The patient then undertook recipes that strictly conformed to the low-fat and MCT diet. After 10 weeks, the results shown in a repeat laboratory examination were normal (Table 1). Results from other laboratory examinations were almost normal. The patient gained weight, and the main complaints disappeared.

## Conclusion

In conclusion, we presented a case in which PIL was suspected which involved edema associated with protein loss. Because IL can be primary or secondary, we first excluded the possibility of secondary IL via tests for rheumatic, neoplastic, and parasitic infection. Then we carried out a capsule endoscopy and double-balloon endoscopy-assisted biopsy. The pathologic analysis of small intestinal tissue showing edema in the submucosa and lymphangiectasia confirmed the diagnosis of IL. The diagnosis of PIL was finally established after excluding the secondary pathogeny. A positive clinical response to the special diet therapy of low-fat and MCTs further confirmed the diagnosis of PIL.

## Consent

Written informed consent was obtained from the patient’s legal guardian for publication of this manuscript and accompanying images. A copy of the written consent is available for review by the Editor-in-Chief of this journal.

## Competing interests

The authors declare that they have no competing interests.

## Authors’ contributions

YL and TY wrote the paper; XYQ and LNZ confirmed the pathologic diagnosis; TY performed the double-balloon enteroscopy; QKC and TY interpreted the patient data regarding PIL and cured the patient. All authors read and approved the final manuscript.
